# Prevalence of alcohol consumption and awareness of consumption guidelines: A population-based study in Geneva, Switzerland

**DOI:** 10.1016/j.pmedr.2026.103415

**Published:** 2026-02-15

**Authors:** Roxane Dumont, Hélène Baysson, Shannon Mechoullam, Céline Mettraux, Silvia Stringhini, Idris Guessous, Mayssam Nehme

**Affiliations:** a Division of Primary Care Medicine, Geneva University Hospitals, Geneva, Switzerland; b School of Population and Public Health and Edwin S.H. Leong Centre for Healthy Aging, Faculty of Medicine, University of British Columbia, Vancouver, Canada; c Department of Health and Community Medicine, Faculty of Medicine, University of Geneva, Geneva, Switzerland

**Keywords:** Alcohol, Guidelines, Risk awareness, Public health, Epidemiology, Primary care

## Abstract

**Objective:**

Alcohol consumption remains a major preventable cause of morbidity and mortality. In Switzerland, approximately 1500 annual deaths are attributable to alcohol. This study aimed to assess awareness of national low-risk drinking guidelines and to estimate the proportion of adults exceeding them in Geneva, according to sociodemographic and health-related factors.

**Methods:**

In 2025, 7509 adults from the Specchio population-based cohort were invited to complete a questionnaire on alcohol consumption, knowledge of Swiss guidelines, and risk perception. Exceeding guidelines was defined as >2 drinks/day for men, >1 for women, or fewer than two alcohol-free days per week. Descriptive analyses and sex-stratified logistic regressions examined associations with sociodemographic, behavioral, and health variables.

**Results:**

Among 4274 respondents (mean age 51.5 years; 59.8% women), 88.3% reported alcohol use, among which 53.5% exceeded national guidelines. Women more frequently surpassed daily limits, whereas men and older adults more often failed to maintain alcohol-free days. Exceeding guidelines was associated with younger age, higher education, favorable financial situation, and substance use. Only 49.1% accurately identified the full guidelines, and 29.1% recognized cancer-related risks.

**Conclusions:**

Nearly half of adults in Geneva exceed national drinking recommendations, underscoring the need for improved public communication and targeted preventive strategies in primary care.

## Highlights

• Over half (53.3%) of Geneva adults exceed national alcohol guidelines.
• Women exceed daily limits more than men (62.4% vs 39.6%).
• Only 49.1% knew complete guidelines; 29.1% recognized cancer risks.
• Higher education and substance use predict guideline exceedance.
• Clearer public health messaging on alcohol risks and guidelines are needed.

## 1 Introduction

Alcohol is a leading contributor to global disease burden and premature mortality, responsible for an estimated 2.6 million deaths worldwide in 2019 (World Health Organization, 2024). Its harms stem mainly from cancers, liver and cardiovascular diseases, injuries in younger populations, and mental health disorders (Anderson et al., 2023; Addiction Suisse, 2025; INSERM and Institut National de la santé et de la Recherche médiacle, 2022; OFSP, Office fédéral de santé publique, 2020; Chikritzhs and Livingston, 2021; Suter et al., 2023). In Switzerland, alcohol accounts for about 1500 deaths annually among adults aged 15–74 (Suter et al., 2023).

To guide consumers and limit health hazards associated with alcohol consumption, many countries—including Australia, the United Kingdom, Canada, and France (National Health and Medical Research Council, 2020; Belisario et al., 2025; Santé publique France, and Santé publique France, Institut national du cancer, 2017; Muradbegovic and Favrod-Coune, 2023) have recently lowered recommended alcohol consumption limits for the general population. In Switzerland, the Federal Commission for Alcohol Problems (CFAL) set guidelines on low-risk drinking in 2018 based on scientific evidence, recommending no more than two standard drinks per day for men, one for women, and at least two alcohol-free days per week to reduce health risks (Commission fédérale pour les problèmes liés à l'alcool (CFAL), 2018), where one standard drink contains 10–12 g of pure alcohol, consistent with most European countries (Bae et al., 2022).

In Geneva, over 80% of adults report regular alcohol consumption in the past year, with nearly half (48%) drinking weekly and 11% drink daily, both patterns being more common among men and older adults (Gerber, 2025). Previous work from our group showed that men with higher socioeconomic status (SES) were more likely to engage in hazardous drinking, while an inverse but weaker SES gradient was observed among women (Sandoval et al., 2019). Despite several alcohol control laws implemented in the past two decades (Dumont et al., 2017), long-term monitoring (1997–2019) indicated limited equity effects, highlighting the need for more targeted prevention strategies. In Switzerland, alcohol control laws are set federally but implemented at cantonal and municipal levels.

The COVID-19 pandemic may have further altered alcohol use by disrupting social behaviors and shifting consumption patterns. Early evidence suggests stable home drinking, reduced consumption in public settings, and increased risk among vulnerable subgroups (Labhart et al., 2022).

While alcohol consumption is well documented globally and in Switzerland (Bae et al., 2022; Sandoval et al., 2019; Dumont et al., 2017; Federal Statistical Office (OFS), 2025), population-level data on public awareness of alcohol-related health risks, guideline knowledge, and exceedance rates remain limited. Previous work focused on consumption patterns or hazardous drinking without addressing public understanding. Such data are essential to evaluate guideline implementation and to guide public health and primary care prevention strategies in both international and local contexts.

### 1.1 Objectives

This population-based study from Geneva (Switzerland) aims to inform local prevention strategies by evaluating awareness of national drinking guidelines, quantifying sex-stratified prevalence of guidelines exceedance, and analyzing associations with sociodemographic and health factors, alongside general awareness of alcohol consumption risks.

## 2 Methods

### 2.1 Study design and population

This study is based on the Specchio cohort, a population-based digital cohort established in December 2020 in Geneva, Switzerland (Baysson et al., 2022). The cohort originated as the “Specchio-COVID19” study in December 2020. In 2023, the study expanded its scope to become a longitudinal assessment of the multifaced of health of the Geneva population. Inclusion criteria were age 18 years and older and living in the Canton of Geneva. Participants accessed the Specchio-Hub platform (www.specchio-hub.ch) via smartphone, laptop, or desktop to complete questionnaires. Following baseline enrollment, participants receive up to four annual thematic questionnaires (15–20 min each) covering multiple health dimensions including mental and physical well-being, occupational health, nutrition, physical activity, substance use, and sleep habits. The study was approved by the Geneva Cantonal Ethics Committee for Research (Number 2020–00881 and Number 2022–02006).

In this analysis, participants completed a thematic questionnaire administered in February 2025 and specifically designed to assess alcohol habits (Supplementary Table S1).

### 2.2 Measures

Participants were asked if they consumed alcohol over the last 7 days and, if so, to report the number of drinks consumed per day for each type of alcoholic beverage (beer or cider, wine or champagne, spirits, aperitifs, premixed drinks, and cocktails). By definition (Commission fédérale pour les problèmes liés à l'alcool (CFAL), 2018; Federal Statistical Office (OFS), 2025), a participant exceeded the consumption guidelines in the last seven days if:

• They reported consuming more than two drinks per day (men) or more than one drink per day (women) at least once in the past seven days,
• Or if they consumed alcohol on more than five days in the past seven days.

Individuals who did not exceed the threshold on either dimension, were considered as not having exceeded the guidelines. Each of the two guidelines was also examined separately. Alcohol consumption was measured using the validated Alcohol Use Disorders Identification Test-Consumption (AUDIT-C) questionnaire, assessing quantity and frequency over 12 months (Bush et al., 1998). This shorter version was selected to reduce survey length while maintaining validation. Additional variables included alcohol consumption last week, 12-month abstinence status, lifetime alcohol use disorder (personal or among close relatives) and binge drinking episodes (≥five drinks in a single occasion during the past month).

Participants' knowledge of alcohol guidelines was assessed through two questions: “In your opinion, what is the maximum number of alcoholic drinks that should not be exceeded in one day to limit health risks?” and “How many days per week can one drink alcohol without taking too many health risks?” Risk perceptions were evaluated using five statements about wine benefits, cardiovascular effects, cancer risks, and alcohol-free days, with response options: “Strongly agree,” “Agree,” “Disagree,” “Strongly disagree,” and “I don't know” (Table S1).

Covariates included age, sex, and education level, nationality, financial situation, and living situation, which are valid proxy for socioeconomic status (Galobardes et al., 2006). Work-related variables included occupation and employment status. Substance use behaviors included smoking status (current consumption with the median number of daily cigarette consumption), e-cigarette use (current consumption), and regular drug use (regular use of cannabis, cocaine, amphetamines, ecstasy/methylenedioxymethamphetamine (MDMA) or other substances). Healthcare access was assessed through self-reported healthcare forgoing due to financial constraints in the past 12 months. Health measures included self-reported physical and mental health status, assessed with the question “Currently, how do you rate your physical and mental health?” using a 5-point Likert scale (very good, good, average, poor, very poor) and subsequently dichotomized (good/very good vs. average/poor/very poor) (De Bruin et al., 1996), and Body Mass Index category defined by World Health Organization classification (Table S1).

### 2.3 Statistical analysis

Prevalence estimates with 95% confidence intervals (CI) were weighted to match the Geneva cantonal population structure for age, sex, and education level, using reference data from the Geneva Office of Statistics from 2023 (Ellis et al., 2018). Table S2 presents differences in age, sex, and education between the Geneva population and the study sample. Adjusted logistic regression analyses were conducted to estimate the associations between socioeconomic, behavioral, health factors and exceeding alcohol consumption guidelines. Analyses were stratified by sex to identify sex-specific risk factors. Age-, sex-, and education- adjusted odds ratios were presented as aOR with 95% CI.

All analyses were conducted using R version 4.4.0 (De Bruin et al., 1996).

## 3 Results

### 3.1 Study population

The questionnaire was sent to 7509 individuals enrolled in the Specchio cohort. After two reminders, 4274 participants completed the questionnaire (57% participation). Participants had a mean age of 51.5 years, (standard deviation SD 12.9; range 18–97), 59.8% were women, and 66.8% had attained higher education (Table 1). Compared with non-participants, respondents were more likely to be women, older, Swiss nationals, wealthier, and non-smokers than non-participants, with no baseline alcohol consumption differences (Supplementary Table S2).

**Table 1:** Distribution of baseline characteristics of adult participants in the Specchio study in Geneva, Switzerland, 2025 (*n* = 4274).

Characteristic	Overall Number of participants *N* = 4274	Guidelines Respected *N* = 2319	Guidelines Exceeded *N* = 1955	*p*-value
**Sociodemographics**				
Age years (Median and interquartile range)	52.0 (43.0–60.0)	53.0 (44.0–62.0)	50.0 (42.0–59.0)	<0.01
	N(%)	N(%)	N(%)	
Age group				<0.01
18–39	769 (18.0%)	380 (16.4%)	389 (19.9%)	
40–64	2781 (65.1%)	1485 (64.0%)	1296 (66.3%)	
65+	723 (16.9%)	454 (19.6%)	269 (13.8%)	
Sex				<0.01
Female	2556 (59.8%)	1224 (52.8%)	1332 (68.1%)	
Male	1716 (40.1%)	1093 (47.1%)	623 (31.9%)	
Other	2 (0.0%)	2 (0.1%)	0 (0.0%)	
Education level (highest achieved)				0.22
Primary (compulsory schooling)	117 (2.7%)	72 (3.1%)	45 (2.3%)	
Secondary (apprenticeship/high school)	1292 (30.2%)	695 (30.0%)	597 (30.5%)	
Tertiary (university)	2853 (66.8%)	1543 (66.5%)	1310 (67.0%)	
Other	12 (0.3%)	9 (0.4%)	3 (0.2%)	
Nationality				0.04
Swiss	3462 (81.0%)	1852 (79.9%)	1610 (82.4%)	
Non-Swiss	812 (19.0%)	467 (20.1%)	345 (17.6%)	
Living situation (household type) *n* = 4253				0.10
Couple with children (own or partner's)	1824 (42.7%)	1003 (43.3%)	821 (42.0%)	
Couple without children	1210 (28.3%)	651 (28.1%)	559 (28.6%)	
Single parent with children	308 (7.2%)	148 (6.4%)	160 (8.2%)	
Living alone	689 (16.1%)	390 (16.8%)	299 (15.3%)	
Cohabiting with others	200 (4.7%)	108 (4.7%)	92 (4.7%)	
Other	10 (0.2%)	5 (0.2%)	5 (0.3%)	
**Work**				
Occupation category (socio-professional classification) *n* = 4271				0.047
Professional-Managers	1399 (32.8%)	765 (33.0%)	634 (32.4%)	
Higher-grade white-collar workers	1195 (28.0%)	636 (27.5%)	559 (28.6%)	
Lower grade white-collar workers	1046 (24.5%)	546 (23.6%)	500 (25.6%)	
Independent workers	100 (2.3%)	69 (3.0%)	31 (1.6%)	
Blue collar workers	338 (7.9%)	192 (8.3%)	146 (7.5%)	
Not concerned	119 (2.8%)	69 (3.0%)	50 (2.6%)	
Other	74 (1.7%)	39 (1.7%)	35 (1.8%)	
Work situation (current employment status)				<0.01
Salaried	2747 (64.3%)	1446 (62.4%)	1301 (66.5%)	
Retired	788 (18.4%)	497 (21.4%)	291 (14.9%)	
Freelance/sole trader	315 (7.4%)	157 (6.8%)	158 (8.1%)	
Homemaker	142 (3.3%)	74 (3.2%)	68 (3.5%)	
Student/training	119 (2.8%)	55 (2.4%)	64 (3.3%)	
Unemployed	108 (2.5%)	57 (2.5%)	51 (2.6%)	
Long-term disability	36 (0.8%)	25 (1.1%)	11 (0.6%)	
Other	19 (0.4%)	8 (0.3%)	11 (0.6%)	
**Socioeconomic**				
Financial situation (self-assessment)				0.02
Average to poor	611 (14.3%)	354 (15.3%)	257 (13.1%)	
Good or Very Good	3475 (81.3%)	1850 (79.8%)	1625 (83.1%)	
No answer	188 (4.4%)	115 (5.0%)	73 (3.7%)	
Subsidies (social assistance recipient)				0.54
No	3936 (92.1%)	2146 (92.5%)	1790 (91.6%)	
Yes	220 (5.1%)	113 (4.9%)	107 (5.5%)	
I don't know or prefer not to answer	118 (2.8%)	60 (2.6%)	58 (3.0%)	
**Health**				
Has forgone healthcare for financial reasons *n* = 2983	358 (12.1%)	201 (12.3%)	157 (11.8%)	0.61
Body Mass Index category (World Health Organization classification)				0.52
Underweight	128 (3.0%)	73 (3.1%)	55 (2.8%)	
Normal weight	2494 (58.4%)	1329 (57.3%)	1165 (59.7%)	
Overweight	1210 (28.3%)	675 (29.1%)	535 (27.4%)	
Obese	440 (10.3%)	242 (10.4%)	198 (10.1%)	
Mental health status (self-assessment)				>0.9
Good	3734 (87.4%)	2027 (87.4%)	1707 (87.3%)	
Average to poor	540 (12.6%)	292 (12.6%)	248 (12.7%)	
Physical health status (self-assessment)				0.01
Good	3859 (90.3%)	2069 (89.2%)	1790 (91.6%)	
Poor	415 (9.7%)	250 (10.8%)	165 (8.4%)	
**Substance Use**				
Smoking status *n* = 4273				<0.01
Current smokers	644 (15.1%)	261 (11.3%)	383 (19.6%)	
Daily number of cigarettes (Median IQR)	10 (5–20)	10 (5–20)	10 (5–20)	0.21
Former smokers	1280 (29.9%)	589 (25.4%)	691 (35.3%)	
Never smokers	2349 (55.0%)	1469 (63.3%)	880 (45.0%)	
*E*-cigarette use (current consumption) *n* = 3724	160 (3.7%)	55 (2.4%)	105 (5.4%)	<0.01
Regular drug use (occasionally, weekly, or daily) *n* = 2956	125 (4.2%)	47 (2.9%)	78 (5.8%)	<0.01

**Footnote:** This table represents the distribution of baseline characteristics of adult participants in the Specchio study in Geneva, Switzerland, 2025 (n = 4274) by adherence to alcohol guidelines. The groups are separated between having respected or exceeded the guidelines Swiss alcohol consumption guidelines include ≤1 drink per day for women, ≤2 drinks per day for men, and having ≥2 alcohol-free days per week, chi-squared test with Rao & Scott's second-order correction. Variables defined as: age group (18–39, 40–64, 65+ years); sex (male, female or other); education (primary, secondary, tertiary); nationality (Swiss vs. non-Swiss); living situation (living alone, couple without/with children, single parent). Work occupation was defined as (Professional-Managers, Higher-grade white-collar workers, Lower grade white-collar workers, Independent workers, Blue collar workers, Not concerned, Other). Work situation was defined as the current employment status (Salaried, Retired, Freelance/sole trader, Homemaker, Student/training, Unemployed, Long-term disability, Other). Financial situation was defined with a self-assessment: “Average to Poor” when participants self-reported they cannot meet needs or covers basic needs; “Good/Very Good” when participants self-reported they can cover needs with ability to save. Subsidies was defined as “Yes” if participants receive subsidies for their healthcare insurance (yes; no; I don't know or prefer not to answer). Forgoing healthcare was defined as self-reported healthcare avoidance due to financial constraints in past 12 months (yes/no). Body Mass Index category (World Health Organization classification) was defined as (Underweight, Normal weight, Overweight, Obese). Mental health status was self-assessed as *Good*, or “Average to poor”. Physical health status was self-assessed as “Good”, or “Poor”. Smoking status was defined as (current vs. non-smoker); e-cigarette use was defined as (current vs. none); drug use was defined as “regular” when participants reported using drug use occasionally, weekly or daily.

### 3.2 Prevalence of alcohol consumption and exceeding guidelines

• Out of the 4274 participants, 11.77% reported being abstinent and 88.33% reported drinking in the past 12 months. The prevalence of exceeding alcohol guidelines in the general population was 43.80% (95% CI 41.10, 46.60).
• Among drinkers (*n* = 3747), the post-stratified prevalence was 79.94% (95% CI 78.52, 81.38) for any alcohol consumption in the past seven days and 27.84% (95% CI 26.32, 29.42) for risky alcohol use according to the Alcohol Use Disorders Identification Test-Consumption (AUDIT-C) (Table 2). Additionally, 53.51% (95% CI 51.81, 55.21) of alcohol drinkers exceeded the alcohol consumption guidelines. Specifically, 49.73% exceeded daily drinking limits, 7.12% consumed alcohol on more than five days per week, and 4.06% (95% CI 3.47, 4.74) did not meet both guidelines simultaneously. Additionally, 23.71% (95% CI 22.27, 25.21) reported at least one episode of excessive alcohol consumption in the last month (Table 2).

**Table 2:** Prevalence of alcohol-related behaviors estimated using post-stratification for age, sex and education of adult participants of the Specchio study in Geneva, Switzerland, 2025 (*n* = 4274).

Characteristic	Response	Overall % [95% CI]^1,2^	Sex		Age groups			
			Female % [95% CI]	Male % [95% CI]	18–24% [95% CI]	25–44% [95% CI]	45–64% [95% CI]	65+ % [95% CI]
**Alcohol abstinence (n = 4274)**								
	No	88.33 [85.22, 90.87]	82.71 [79.21, 85.70]	83.60 [77.70, 88.20]	86.70 [undefined]	88.29 [85.24, 90.79]	82.72 [79.10, 85.72]	83.60 [77.72, 88.20]
	Yes	11.77 [9.16, 14.80]	17.39 [14.30, 20.82]	16.40 [11.80, 22.30]	13.30 [0,0]	11.71 [9.16, 14.80]	17.28 [14.31, 20.81]	16.40 [11.86, 22.31]
**Alcohol consumption last week (n = 3747)**								
	No	20.06 [18.72, 21.48]	24.30 [22.42, 26.28]	13.52 [11.84, 15.41]	32.47 [22.67, 44.09]	23.02 [20.41, 25.85]	18.65 [17.01, 20.40]	15.50 [12.81, 18.64]
	Yes	79.94 [78.52, 81.28]	75.70 [73.72, 77.58]	86.48 [84.59, 88.16]	67.53 [55.91, 77.33]	76.98 [74.15, 79.59]	81.35 [79.60, 82.99]	84.50 [81.36, 87.19]
**Respects daily drinking limits (n = 3747)**								
	No	50.27 [48.57, 51.97]	60.69 [58.51, 62.84]	34.18 [31.74, 36.72]	60.54 [48.99, 71.01]	56.72 [53.52, 59.87]	48.72 [46.53, 50.90]	32.41 [28.79, 36.25]
	Yes	49.73 [48.03, 51.43]	39.31 [37.16, 41.49]	65.82 [63.28, 68.26]	39.46 [28.99, 51.01]	43.28 [40.13, 46.48]	51.28 [49.10, 53.47]	67.59 [63.75, 71.21]
**≥2 alcohol-free days per week (n = 3747)**								
	No	7.12 [6.35, 7.98]	5.41 [4.54, 6.45]	9.76 [8.39, 11.32]	–	3.49 [2.49, 4.87]	7.82 [6.72, 9.07]	18.36 [15.50, 21.61]
	Yes	92.36 [91.46, 93.16]	94.28 [93.22, 95.18]	89.38 [87.76, 90.82]	100.00 [undefined]	96.18 [94.75, 97.23]	91.67 [90.38, 92.80]	80.23 [76.88, 83.19]
**Guidelines exceeded (OR) (n = 3747)**								
	No	46.49 [44.79, 48.19]	37.57 [35.45, 39.75]	60.32 [57.72, 62.85]	39.46 [28.99, 51.01]	41.78 [38.66, 44.98]	48.20 [46.01, 50.39]	56.14 [52.14, 60.06]
	Yes	53.51 [51.81, 55.21]	62.43 [60.25, 64.55]	39.68 [37.15, 42.28]	60.54 [48.99, 71.01]	58.22 [55.02, 61.34]	51.80 [49.61, 53.99]	43.86 [39.94, 47.86]
**Guidelines exceeded (AND) (n = 3747)**								
	No	95.94 [95.26, 96.53]	96.24 [95.35, 96.96]	95.49 [94.32, 96.43]	100.00 [undefined]	97.94 [96.82, 98.67]	95.08 [94.04, 95.95]	92.61 [90.25, 94.44]
	Yes	4.06 [3.47, 4.74]	3.76 [3.04, 4.65]	4.51 [3.57, 5.68]	[undefined]	2.06 [1.33, 3.18]	4.92 [4.05, 5.96]	7.39 [5.56, 9.75]
**At-risk drinking according to Alcohol Use Disorders Identification Test-Consumption (AUDIT-C) (n = 3747)**								
	No	72.16 [70.58, 73.68]	64.13 [61.95, 66.26]	84.55 [82.53, 86.37]	73.84 [62.62, 82.62]	68.07 [64.99, 71.00]	72.93 [70.94, 74.83]	84.17 [81.01, 86.89]
	Yes	27.84 [26.32, 29.42]	35.87 [33.74, 38.05]	15.45 [13.63, 17.47]	26.16 [17.38, 37.38]	31.93 [29.00, 35.01]	27.07 [25.17, 29.06]	15.83 [13.11, 18.99]
**Binge drinking episodes (n = 3747)**								
	No	76.29 [74.79, 77.73]	73.71 [71.68, 75.65]	80.27 [78.05, 82.32]	74.80 [63.50, 83.51]	70.73 [67.73, 73.57]	77.97 [76.11, 79.72]	89.12 [86.40, 91.35]
	Yes	23.71 [22.27, 25.21]	26.29 [24.35, 28.32]	19.73 [17.68, 21.95]	25.20 [16.49, 36.50]	29.27 [26.43, 32.27]	22.03 [20.28, 23.89]	10.88 [8.65, 13.60]
**Alcohol use disorder (n = 4274)**								
	No	68.72 [66.40, 70.90]	62.70 [59.20, 66.20]	75.30 [72.40, 77.90]	81.40 [71.40, 88.50]	69.30 [65.90, 72.50]	63.50 [60.20, 66.70]	69.70 [62.90, 75.70]
	Yes, for myself	2.66 [2.17, 3.27]	2.10 [1.54, 2.86]	3.53 [2.69, 4.63]	2.30 [0.58, 8.69]	2.75 [1.88, 4.00]	2.67 [2.05, 3.47]	2.31 [1.39, 3.81]
	Yes, for a close person	32.36 [30.78, 33.98]	37.01 [34.87, 39.19]	25.18 [22.97, 27.54]	25.19 [16.59, 36.32]	32.54 [29.60, 35.63]	33.71 [31.68, 35.81]	24.03 [20.81, 27.58]
**Knowledge: Men's daily limit (≤2 drinks) (n = 4274)**								
	No	29.96 [28.44, 31.53]	30.63 [28.62, 32.71]	28.94 [26.64, 31.35]	39.50 [29.03, 51.03]	27.00 [24.23, 29.96]	30.07 [28.11, 32.11]	40.75 [36.91, 44.72]
	Yes	67.68 [66.08, 69.24]	67.12 [65.00, 69.16]	68.55 [66.08, 70.92]	57.53 [46.02, 68.29]	70.84 [67.82, 73.68]	67.58 [65.50, 69.59]	56.10 [52.12, 60.01]
	Don't know	2.36 [1.90, 2.92]	2.26 [1.71, 2.97]	2.51 [1.80, 3.50]	2.97 [0.74, 11.10]	2.17 [1.43, 3.27]	2.35 [1.77, 3.12]	3.14 [2.02, 4.85]
**Knowledge: Women's daily limit (≤1 drink) (n = 4274)**								
	No	43.92 [42.24, 45.61]	42.91 [40.72, 45.13]	45.48 [42.89, 48.09]	48.38 [37.19, 59.73]	39.54 [36.45, 42.73]	44.69 [42.52, 46.87]	56.96 [52.99, 60.84]
	Yes	53.36 [51.66, 55.05]	55.16 [52.93, 57.36]	50.59 [47.97, 53.21]	48.66 [37.45, 60.00]	58.18 [54.99, 61.31]	52.53 [50.34, 54.70]	38.96 [35.16, 42.89]
	Don't know	2.72 [2.23, 3.30]	1.93 [1.42, 2.61]	3.93 [3.04, 5.07]	2.97 [0.74, 11.10]	2.27 [1.51, 3.40]	2.79 [2.15, 3.61]	4.09 [2.79, 5.95]
**Knowledge: Weekly limit (≤5 drinking days per week) (n = 4274)**								
	Don't know	8.61 [7.73, 9.57]	9.36 [8.19, 10.67]	7.45 [6.21, 8.91]	4.55 [1.48, 13.15]	6.09 [4.75, 7.77]	9.64 [8.43, 11.00]	13.02 [10.57, 15.94]
	No	4.12 [3.53, 4.82]	3.00 [2.34, 3.83]	5.87 [4.81, 7.15]	[undefined]	2.75 [1.87, 4.04]	4.18 [3.39, 5.13]	9.74 [7.66, 12.32]
	Yes	87.27 [86.14, 88.32]	87.65 [86.17, 88.99]	86.68 [84.87, 88.31]	95.45 [86.85, 98.52]	91.16 [89.19, 92.80]	86.18 [84.61, 87.61]	77.24 [73.76, 80.38]
**Overall knowledge of alcohol guidelines (n = 4274)**								
	No	44.71 [41.80, 47.62]	44.38 [40.81, 48.17]	44.08 [40.32, 49.64]	42.44 [28.9, 57.0]	37.89 [33.10, 42.92]	45.10 [41.71, 48.64]	57.50 [50.42, 63.47]
	Yes	49.09 [46.23, 50.21]	49.22 [45.62, 52.86]	49.02 [44.31, 53.90]	49.25 [34.3, 64.4]	57.51 [52.42, 62.31]	47.91 [44.29, 51.50]	36.50 [30.41, 43.20]
	Don't know	6.20 [4.91, 7.81]	6.30 [4.91, 8.26]	6.00 [4.01, 9.20]	8.31 [undefined]	4.67 [3.52, 6.02]	7.00 [5.41, 9.12]	6.52 [4.23, 10.11]

**Footnote:** This table presents weighted prevalence estimates for alcohol abstinence, consumption behaviors in the past week, adherence to daily and weekly drinking limits, at-risk drinking indicators, alcohol use disorder, and knowledge of Swiss drinking recommendations among adult participants in the Specchio study in Geneva, Switzerland, 2025 (n = 4274). All prevalence were estimated using post-stratification for age, sex and education of the general population in Geneva, Switzerland (year 2023). Alcohol consumption recommendation in Switzerland is defined as 1 drink or less per day for women, 2 drinks or less per day for men, and having 2 or more alcohol-free days per week. Binge drinking defined ≥5 drinks on a single occasion in the past month. Alcohol consumption was measured using the validated Alcohol Use Disorders Identification Test-Consumption (AUDIT-C) questionnaire, assessing quantity and frequency over 12 months. This shorter version was selected to reduce survey length while maintaining validation. The AUDIT-C score was calculated by summing three components (frequency of drinking, typical quantity consumed, and frequency of heavy episodic drinking), each scored 0–4 points, with sex-specific thresholds applied to classify risk (≥4 for males, ≥3 for females). (1) n (unweighted) (%); Mean (SD) (2) CI = Confidence Interval (3) chi-squared test with Rao & Scott's second-order correction; Wilcoxon rank-sum test for complex survey samples, testing across stratification.
• Women were significantly more likely than men to go beyond daily drinking, while men were more likely to drink on more than five days per. Across age groups, middle-aged adults showed the highest rates of exceeding daily limits, while older adults (65 years and older considered as 65+) were most likely to drink on more than five days per week (Table 2).

### 3.3 Prevalence of awareness of guidelines and risks of alcohol consumption

Overall, 49.09% (95% CI 46.23, 52.21) of participants correctly identified the complete set of alcohol consumption guidelines (Table 2). Knowledge of the complete guidelines did not differ significantly between men and women. However, there were significant age-related differences in guideline knowledge, with younger adults aged 25–44 showing the highest level of correct knowledge (57.51%, 95% CI 52.42, 62.31), followed by those aged 45–64 (47.91%, 95% CI 44.29, 51.50), and older adults aged 65+ having the least knowledge (36.50%, 95% CI 30.41, 43.20)(Table 2).

**Fig. 1:**
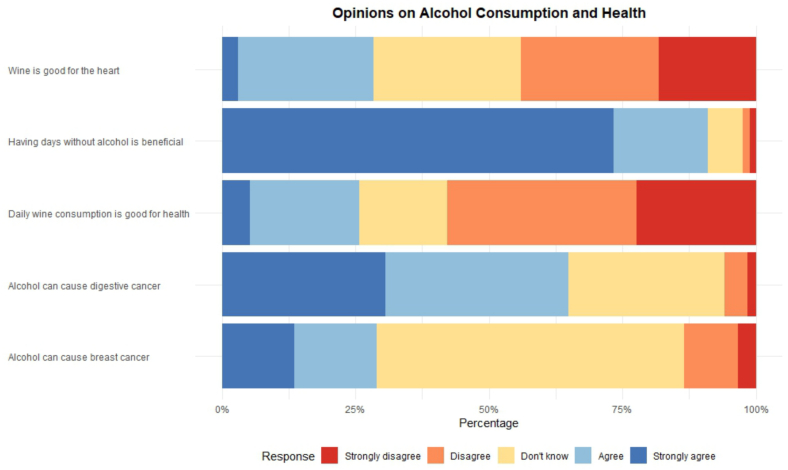
Self-reported perceived health risks associated with alcohol consumption, among adult participants in the Specchio study in Geneva, Switzerland, 2025 (n = *n* = 4274). **Footnote**: Participants were asked to rate their agreement with statements about alcohol and health on a 5-point scale (strongly agree, agree, don't know, disagree, strongly disagree). The figure shows the distribution of responses for each statement, with percentages representing the proportion of participants in each response category. Blue shades indicate agreement (strongly agree and agree), while red/orange shades indicate disagreement (disagree and strongly disagree).

Overall, 62.41% agreed or somewhat agreed that alcohol increases the risk of digestive cancers. Only 29.12% agreed or somewhat agreed that alcohol increases the risk of breast cancer, with 57.34% being unsure. About 30.23% perceived wine as beneficial for cardiovascular health, whereas 85% agreed that having alcohol-free days is beneficial (Fig. 1).

### 3.4 Factors associated with exceeding alcohol consumption guidelines

**Fig. 2:**
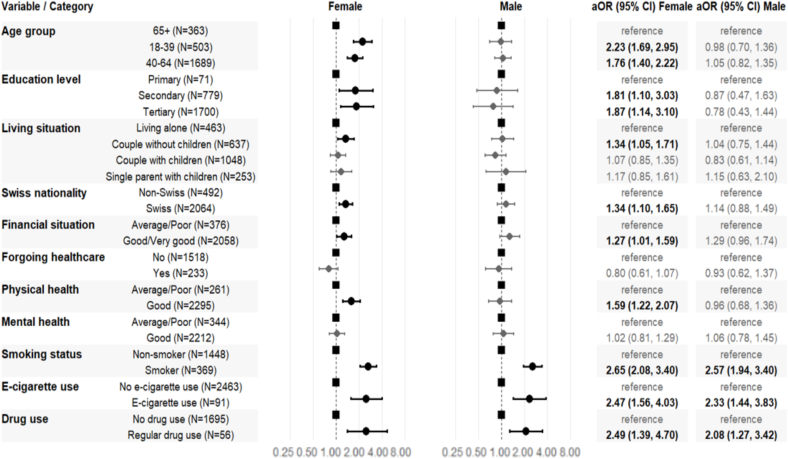
Sociodemographic, health and behavioral factors associated with exceeding alcohol consumption guidelines by sex among adult participants in the Specchio study in Geneva, Switzerland, 2025 (*n* = 3747). **Footnote**: This forest plot shows adjusted odds ratios (aOR) and 95% confidence intervals (CI) for factors associated with exceeding alcohol guidelines, stratified by sex. Models adjusted for age, sex, and education using complete case analysis. Bold markers indicate statistical significance (*p* < 0.05). Dashed line represents aOR = 1.0. Variables defined as: age group (18–39, 40–64, 65+ years); education (primary, secondary, tertiary); living situation (living alone, couple without/with children, single parent); nationality (Swiss vs. non-Swiss); financial situation was defined with a self-assessment: “Average to Poor” when participants self-reported they cannot meet needs or covers basic needs; “Good/Very Good” when participants self-reported they can cover needs with ability to save; forgoing healthcare was defined as self-reported healthcare avoidance due to financial constraints in past 12 months (yes/no); physical/mental health (average/poor vs. good/very good, self-reported); smoking status (current vs. non-smoker); e-cigarette use (current vs. none); drug use (regular vs. none).

Among women, younger age was associated with exceeding alcohol consumption guidelines (aOR 2.23, 95% CI 1.69, 2.95; and aOR 1.75, 95% CI 1.40, 2.22, respectively for women 18–39 years, and 40–64 years). Socioeconomic factors also played a role: compared to primary education, secondary (aOR 1.81, 95% CI 1.10, 3.03) and tertiary education (aOR 1.87, 95% CI 1.14, 3.10) showed higher odds of exceeding guidelines. Being in a couple without children was associated with higher odds of exceeding alcohol consumption guidelines versus living alone, even after adjustment for age, sex and education (aOR 1.34, 95% CI 1.05, 1.71). Swiss nationality increased the odds of exceeding alcohol consumption guidelines (aOR 1.34, 95% CI 1.10, 1.65), as did having a good or very good financial situation compared to an average to poor (aOR 1.27, 95% CI 1.01, 1.59). Women with good physical health (vs. average to poor) had higher odds of exceeding guidelines (aOR 1.59, 95% CI 1.22, 2.07), mental health status showed no significant association. Substance use behaviors demonstrated strong associations: current smoking (aOR 2.65, 95% CI 2.08, 3.40), e-cigarette use (aOR 2.47, 95% CI 1.56, 4.03), and regular drug use (aOR 2.49, 95% CI 1.39, 4.70) all substantially increased the odds of exceeding alcohol guidelines (Fig. 2).

In contrast to women, among men, age, living situation, nationality, financial status, or physical or mental health were not related to exceeding alcohol consumption guidelines. However, substance use patterns remained strongly associated with exceeding alcohol consumption guidelines with smoking, e-cigarette use, and regular drug use.

### 3.5 Factors associated with lack of awareness of alcohol consumption guidelines

**Fig. 3:**
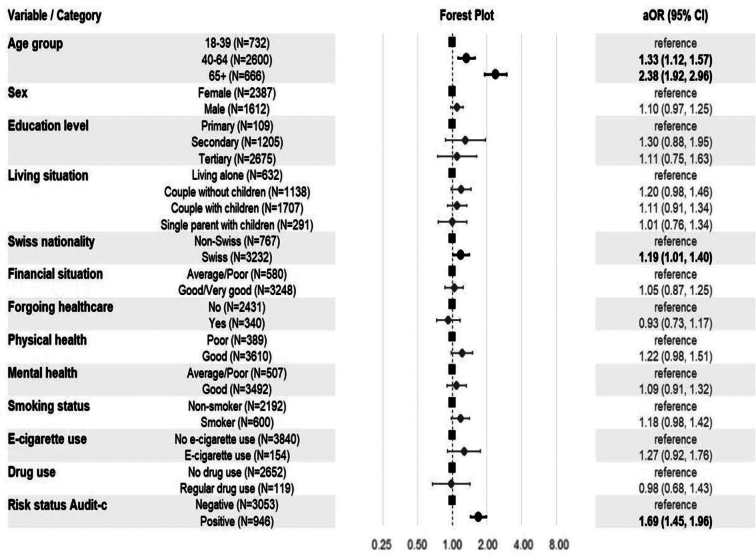
Sociodemographic, health and behavioral factors associated with lack of awareness of Swiss alcohol consumption guidelines among adult participants in the Specchio study in Geneva, Switzerland, 2025 (*n* = 4274). **Footnote**: This forest plot shows adjusted odds ratios (aOR) and 95% confidence intervals (CI) for factors associated with incorrect knowledge of alcohol guidelines. Models adjusted for age, sex, and education using complete case analysis. Bold markers indicate statistical significance (*p* < 0.05). Dashed line represents aOR = 1.0. Variables defined as: age group (18–39, 40–64, 65+ years); education (primary, secondary, tertiary); living situation (living alone, couple without/with children, single parent); nationality (Swiss vs. non-Swiss); financial situation was defined with a self-assessment: “Average to Poor” when participants self-reported they cannot meet needs or covers basic needs; “Good/Very Good” when participants self-reported they can cover needs with ability to save; forgoing healthcare was defined as self-reported healthcare avoidance due to financial constraints in past 12 months (yes/no); physical/mental health (average/poor vs. good/very good, self-reported); smoking status (current vs. non-smoker); e-cigarette use (current vs. none); drug use (regular vs. none). Alcohol consumption was measured using the validated Alcohol Use Disorders Identification Test-Consumption (AUDIT-C) questionnaire, assessing quantity and frequency over 12 months. This shorter version was selected to reduce survey length while maintaining validation. The AUDIT-C score was calculated by summing three components (frequency of drinking, typical quantity consumed, and frequency of heavy episodic drinking), each scored 0–4 points, with sex-specific thresholds applied to classify risk (≥4 for males, ≥3 for females).

When looking at factors associated with lack of awareness of alcohol consumption guidelines (Fig. 3), older individuals showed a higher lack of awareness of alcohol consumption guidelines compared to the youngest age group, with those aged 40–64 (aOR = 1.33, 95% CI 1.12, 1.57) and those aged 65+ (aOR = 2.38, 95% CI 1.92, 2.96). Participants with Swiss nationality also showed slightly higher odds of poor knowledge compared to non-Swiss nationals (aOR = 1.19, 95% CI 1.01, 1.40). Most demographic and health factors showed no significant associations. However, alcohol consumption patterns were strongly predictive of lack of awareness of alcohol consumption guidelines. Participants with positive Alcohol Use Disorders Identification Test-Consumption (AUDIT-C) screening had higher odds of not knowing the alcohol consumption guidelines (aOR = 1.69, 95% CI 1.45, 1.96), while those exceeding the current guidelines had more than double the odds of poor knowledge.

## 4 Discussion

This population-based study suggest that half of the population exceeds the Swiss alcohol consumption guidelines in 2025. Women and younger individuals were more likely to exceed these guidelines, as well as smokers and substance users. Lack of awareness of the alcohol consumption guidelines was higher in older individuals and those who consumed more alcohol.

These results provide updated post-pandemic estimates, complementing our previous findings and revealing no considerable impact of alcohol control laws implemented during this period (Sandoval et al., 2019). From a public health perspective, this underlines the urgency of reinforcing awareness campaigns and systematic screening in primary care to identify risky drinking patterns early.

In our study, women—particularly those with higher educational attainment—were more likely to exceed the alcohol guidelines compared to men, in contrast to findings in other studies (El Haddad et al., 2024; Berry et al., 2007). This discrepancy may partly reflect the stricter Swiss guidelines for women (one drink per day versus two for men), and Geneva's educated, urban population where sex convergence in drinking patterns is increasingly observed among professionals (Donat et al., 2024). Nevertheless, prior research has also reported that women with higher education are more likely to engage in harmful drinking, while this pattern is less consistently observed in men (Grittner et al., 2015). These findings highlight the importance of tailoring prevention campaigns to women beyond just pregnancy.

Younger adults were more likely to exceed daily drink limits (one–two drinks per day), and among 29 to 44 years old, 27% reported binge drinking. While older adults (65+) were less likely to exceed daily limits, they were three times more likely to drink more than five days a week. This is concerning given older adults' increased vulnerability to alcohol-related risks including medication interactions, falls, and exacerbated pre-existing conditions. Targeted messaging should focus on drinking frequency and promoting alcohol-free days.

Exceeding alcohol consumption guidelines was more common among smokers, and individuals with higher socioeconomic status. Higher socioeconomic status individuals tend to exceed alcohol consumption guidelines more frequently, possibly due to broader social networks, fewer financial constraints, and generally better overall health status (INSERM and Institut National de la santé et de la Recherche médiacle, 2022). Exceeding guidelines for alcohol consumption has also been associated with the use of other substances such as tobacco and cannabis (El Haddad et al., 2024). For public health, this suggests the need for integrated lifestyle approaches rather than siloed campaigns, promoting reduced consumption across substances.

Our study also shows that women who rated their health as good or very good were more likely to exceed alcohol consumption guidelines. A similar association has been observed in previous studies (Bae et al., 2022; Lange et al., 2016). One possible explanation is that exceeding alcohol guidelines is not necessarily pathological in the short term, even though it is not recommended. Instead, individuals with poorer health may be less likely to engage in social drinking. However, this should be considered given health selection and healthy user bias, as social drinkers may represent a subgroup with better baseline health and stronger social integration. At the same time, it is important to distinguish this from pathological alcohol use, which in our study was associated with worse self-rated health and aligns with the evidence that sustained harmful consumption negatively impacts health. Of note, our study showed no association between exceeding alcohol consumption guidelines and mental health; this could also be due to the differentiation between pathological alcohol consumption and exceeding the recommendations without engaging in high-risk behavior.

When considering awareness and risk perception, although two-thirds of participants correctly identified national guidelines, results showed notable differences by sex and age. There were also discrepancies between awareness of guidelines, behavior, and knowledge of risks. Only 29% recognized alcohol as a risk factor for breast cancer, and nearly one-third perceived wine as beneficial for cardiovascular health. Such beliefs, together with inconsistent messaging across countries and sex-specific thresholds, may contribute to poor adherence to guidelines. To address this, communication strategies should prioritize clarity, emphasize that no amount of alcohol is risk-free, and explicitly highlight cancer risks. This is particularly important given that 30% believe wine has cardiovascular benefits. Educational information should be disseminated through ongoing media campaigns and social marketing efforts, as well as through innovative channels. Campaigns modeled on successful tobacco control strategies may be required to correct misconceptions and reduce the normalization of alcohol in everyday life. Awareness could shift drinking norms and support clinical interventions in primary care. Longitudinal monitoring will be essential to evaluate whether increased guideline awareness translates into consumption reductions (World Health Organization, 2024; Dumont et al., 2017), as behavioral impact often remains limited without structural interventions.

Beyond reinforcing primary prevention through screening and healthy behaviors, structural measures could include banning online alcohol advertising (OMS, 2024); increasing taxes (Addiction Suisse, 2025); limiting alcohol availability; adding warning labels (Zuckermann et al., 2024); promoting abstinence campaigns such as *Dry January* (de Visser and Nicholls, 2020).

Additionally, sex-differentiated guidelines may hinder message clarity. Countries like France, the UK, Netherlands, and Portugal use unified recommendations. While this simplifies messaging, it may falsely suggest identical health risks for men and women. As the World Health Organization emphasizes, it may be more effective to state that no alcohol amount is entirely risk-free, especially regarding cancer (World Health Organization, 2024; World Health Organization, 2025).

This study includes some limitations. Results were self-reported, which may lead to underestimation of actual consumption (Gerber, 2025). Self-reported alcohol consumption may be inaccurate due to recall bias and social desirability. The seven-day recall period, while improving accuracy, may be short for establishing typical patterns, as individuals experience atypical weeks due to illness or vacation. Additionally, drink quantities used ranges and standard drinks, which may not perfectly reflect actual consumption. Non-response bias is also possible if individuals with specific health behaviors choose not to participate (van Gelder et al., 2010). However, a comparison of respondents and non-respondents in this study showed no significant differences in alcohol use, smoking, or self-rated health at the time of enrolment. Additional limitations include the cross-sectional design, which prevents causal inference, and the Geneva-specific setting, which may limit generalizability to broader Swiss populations*.* Finally, post-stratification adjustment of prevalence rates mitigates underrepresentation of younger individuals and those with lower educational levels.

## 5 Conclusion

Alcohol consumption remains highly prevalent in Switzerland and should be accompanied by health messaging and more awareness of guidelines and increased health risks. Primary care settings represent a crucial point of contact for the early detection of risky alcohol use, enabling healthcare providers to raise awareness, correct misconceptions, and encourage behavioral change. Implementing unified alcohol consumption thresholds—regardless of sex—may enhance message clarity and facilitate integration into routine clinical practice, while targeting groups that are at higher risk.

## Declaration of AI-assisted technologies in the writing process

During the preparation of this work, the authors used Claude generative AI to correct orthography and rephrase text when necessary to improve clarity. After using this tool/service, the authors reviewed and edited the content as needed and take full responsibility for the content of the published article**.**

## CRediT authorship contribution statement

**Roxane Dumont:** Writing – review & editing, Writing – original draft, Methodology, Formal analysis, Conceptualization, Visualization. **Hélène Baysson:** Writing – original draft, Methodology, Conceptualization, Writing – review & editing. **Shannon Mechoullam:** Writing – review & editing, Data curation. **Céline Mettraux:** Writing – review & editing, Data curation. **Silvia Stringhini:** Writing – review & editing, Validation. **Idris Guessous:** Writing – review & editing, Validation, Investigation, Conceptualization, Methodology, Project administration, Supervision. **Mayssam Nehme:** Writing – review & editing, Writing – original draft, Validation, Supervision, Methodology, Formal analysis, Conceptualization, Investigation, Project administration, Visualization.

## Funding

This study is funded by the Cantonal Office of Health in Geneva, Switzerland.

## Declaration of competing interest

The authors declare that they have no known competing financial interests or personal relationships that could have appeared to influence the work reported in this paper.

## Data Availability

Data will be made available on request.

## Appendix A Supplementary data

Supplementary material Image 1
