# Supplementary material for: Prevalence of alcohol consumption and awareness of consumption guidelines: A population-based study in Geneva, Switzerland
Source: Prev Med Rep. 2026 Feb 15;63:103415. doi: 10.1016/j.pmedr.2026.103415 (PMC12938117; doi:10.1016/j.pmedr.2026.103415)
Supplement: Supplementary file 1 — Supplementary material [file mmc1.docx]

**Prevalence of alcohol consumption and awareness of consumption guidelines: a population-based study in Geneva, Switzerland**

**Supplementary material**

**Table S1.** Description of all the variable included in this research

| **Variable** | **Categories/Values** | **Construction Details** |
| --- | --- | --- |
| **SOCIO-DEMOGRAPHIC VARIABLES** |  |  |
| Age at questionnaire | Continuous variable (18-97 years) | Age calculated from date of birth to February 2025 questionnaire completion |
| Age group | 18-39 years / 40-64 years / 65+ years | Categorical groupings based on continuous age variable |
| Sex | Female / Male / Other | Self-reported sex as declared by participant |
| Financial situation | Average to Poor / Good or Very Good / No answer | Self-assessment: "Average to Poor" = cannot meet needs or covers basic needs but vulnerable; "Good/Very Good" = covers needs with ability to save |
| Education level | Primary / Secondary / Tertiary / Other | Highest educational attainment: Primary (compulsory schooling), Secondary (apprenticeship/high school), Tertiary (university) |
| Social assistance | No / Yes / Don't know or prefer not to answer | Current receipt of social assistance or subsidies |
| Living situation | Couple with children / Couple without children / Single parent / Living alone / Cohabiting with others / Other | Current household composition and living arrangement |
| Swiss nationality | Swiss / Non-Swiss | Swiss citizenship status |
| Occupation category | Professional-Managers / Higher-grade white-collar / Lower-grade white-collar / Independent workers / Blue collar / Not concerned / Other | Socio-professional classification based on current or most recent occupation |
| Work situation | Salaried / Retired / Freelance/sole trader / Homemaker / Student/training / Unemployed / Long-term disability / Other | Current employment status |
| **HEALTH VARIABLES** |  |  |
| BMI category | Underweight / Normal weight / Overweight / Obese | World Health Organization classification: <18.5 / 18.5-24.9 / 25.0-29.9 / ≥30.0 kg/m² |
| Mental health status | Good / Average to poor | Self-rated mental health on 5-point Likert scale, dichotomized: "Good" (good/very good) vs "Average to poor" (average/poor/very poor) |
| Physical health status | Good / Poor | Self-rated physical health on 5-point Likert scale, dichotomized: "Good" (good/very good) vs "Poor" (average/poor/very poor) |
| Forgoing healthcare | Yes / No | Self-reported healthcare avoidance due to financial constraints in past 12 months |
| **SUBSTANCE USE VARIABLES** |  |  |
| Smoking status | Current smokers / Former smokers / Never smokers | Current tobacco smoking status with daily cigarette consumption (median, interquartile range) for current smokers |
| E-cigarette use | Yes / No | Current e-cigarette consumption |
| Regular drug use | Yes / No | Occasional, weekly, or daily use of cannabis, cocaine, amphetamines, ecstasy/MDMA, or other substances |
| **ALCOHOL-RELATED VARIABLES** |  |  |
| Alcohol abstinence | Yes / No | Current complete abstinence from alcohol consumption |
| Alcohol consumption last week | No / Yes | Any alcohol consumption in the 7 days prior to questionnaire |
| Respects daily drinking limits | Yes / No | ≤1 drink/day for women, ≤2 drinks/day for men in past 7 days |
| ≥2 alcohol-free days per week | Yes / No | At least 2 days without alcohol consumption in past 7 days |
| Guidelines exceeded (OR) | No / Yes | Exceeded daily limits OR <2 alcohol-free days per week |
| Guidelines exceeded (AND) | No / Yes | Exceeded daily limits AND <2 alcohol-free days per week |
| At-risk drinking (AUDIT-C) | Negative / Positive | AUDIT-C score: Low-risk (0-4) vs Increasing/high-risk (≥5). Validated 3-question screening tool assessing alcohol quantity and frequency over 12 months |
| Alcohol use disorder | No / Yes for myself / Yes for close person / Other / Prefer not to answer | Lifetime exposure to alcohol dependence or alcoholism (personal or family/close contacts) |
| Binge drinking episodes | No / Yes | ≥5 drinks on single occasion in past month |
| **KNOWLEDGE VARIABLES** |  |  |
| Knowledge: Women's daily limit | Yes / No / Don't know | Correct identification of ≤1 drink/day limit for women. Question: "What is the maximum number of alcoholic drinks that should not be exceeded in one day to limit health risks?" |
| Knowledge: Men's daily limit | Yes / No / Don't know | Correct identification of ≤2 drinks/day limit for men |
| Knowledge: Weekly limit | Yes / No / Don't know | Correct identification of ≤5 drinking days per week. Question: "How many days per week can one drink alcohol without taking too many health risks?" |
| Overall knowledge of guidelines | Yes / No / Don't know | Correct knowledge of all three components: women's daily limit, men's daily limit, and weekly frequency limit |

**Notes:**

- One standard drink = 10-12g pure alcohol, consistent with European standards
- Guideline exceedance based on Swiss alcohol consumption recommendations, defined as include ≤1 drink per day for women, ≤2 drinks per day for men, and having ≥2 alcohol-free days per week

**Table S2.** Comparison of sociodemographic characteristics between the Geneva population and adult participants in the Specchio study in Geneva, Switzerland in 2025 (n=4274).

| **Characteristic** | **Geneva population** | **Sample** | **p-value** |
| --- | --- | --- | --- |
| **Sociodemographics** |  |  |  |
| **Sex** |  |  | <0.01 |
| Female | 262,015 (55.0%) | 2,556 (59.8%) |  |
| Male | 214,665 (45.0%) | 1,716 (40.1%) |  |
| **Age group** |  |  | <0.01 |
| 18-39 | 140,481 (29.5%) | 769 (18.0%) |  |
| 40-64 | 201,053 (42.2%) | 2,781 (65.1%) |  |
| 65+ | 135,146 (28.4%) | 723 (16.9%) |  |
| **Education level (highest achieved)** |  |  | <0.01 |
| Primary (compulsory schooling) | 108,935 (22.9%) | 117 (2.7%) |  |
| Secondary (apprenticeship/high school) | 161,362 (33.9%) | 1,292 (30.2%) |  |
| Tertiary (university) | 205,383 (43.1%) | 2,853 (66.8%) |  |
| Other | - | 12 (0.3%) |  |

Age groups were defined as (18-39, 40-64, 65+), Sex was defined as (male, female), Education was defined as highest level of education achieved (primary, secondary, tertiary or other).

**Table S3.** Description of the Specchio cohort (N=7617) and comparison of socio-demographic characteristics between participants (N=4289) and non-participants (N=3220) to the Alcohol questionnaire, February 2025 in Geneva Switzerland

| **Characteristic** | **Overall**  N = 7509*^1^* | **Participants**  N = 4289*^1^* | **Non-participants**  N = 3220*^1^* | **p-value***^2^* |
| --- | --- | --- | --- | --- |
| Sex |  |  |  | <0.01 |
| Female | 4,368 (58.2%) | 2,556 (59.6%) | 1,812 (56.3%) |  |
| Male | 3,119 (41.5%) | 1,716 (40.0%) | 1,403 (43.6%) |  |
| Other | 21 (0.3%) | 16 (0.4%) | 5 (0.2%) |  |
| Age at questionnaire submission | 50.4 (13.8) | 51.6 (12.9) | 48.9 (14.7) | <0.01 |
| Age group |  |  |  | <0.01 |
| 18-39 | 1,668 (22.2%) | 771 (18.0%) | 897 (27.9%) |  |
| 40-64 | 4,599 (61.3%) | 2,792 (65.1%) | 1,807 (56.1%) |  |
| 65+ | 1,239 (16.5%) | 724 (16.9%) | 515 (16.0%) |  |
| Education level |  |  |  | <0.01 |
| Primary | 279 (3.7%) | 117 (2.7%) | 162 (5.0%) |  |
| Secondary | 2,236 (29.8%) | 1,296 (30.2%) | 940 (29.2%) |  |
| Tertiary | 4,965 (66.2%) | 2,863 (66.8%) | 2,102 (65.3%) |  |
| Other | 25 (0.3%) | 12 (0.3%) | 13 (0.4%) |  |
| Financial situation |  |  |  | <0.01 |
| Average to poor | 1,207 (16.1%) | 613 (14.3%) | 594 (18.4%) |  |
| Good or Very Good | 5,916 (78.8%) | 3,486 (81.3%) | 2,430 (75.5%) |  |
| No answer | 380 (5.1%) | 189 (4.4%) | 191 (5.9%) |  |
| Unknown | 6 (0.1%) | 1 (0.0%) | 5 (0.2%) |  |
| Swiss nationality |  |  |  | <0.01 |
| True | 5,870 (78.2%) | 3,474 (81.0%) | 2,396 (74.5%) |  |
| False | 1,634 (21.8%) | 814 (19.0%) | 820 (25.5%) |  |
| Work situation |  |  |  | <0.01 |
| Salaried | 4,845 (64.5%) | 2,757 (64.3%) | 2,088 (64.9%) |  |
| Retired | 1,321 (17.6%) | 789 (18.4%) | 532 (16.5%) |  |
| Freelance/sole trader | 498 (6.6%) | 316 (7.4%) | 182 (5.7%) |  |
| Homemaker | 252 (3.4%) | 143 (3.3%) | 109 (3.4%) |  |
| Student/training | 270 (3.6%) | 120 (2.8%) | 150 (4.7%) |  |
| Unemployed | 219 (2.9%) | 108 (2.5%) | 111 (3.4%) |  |
| Infirm/long term illness | 69 (0.9%) | 36 (0.8%) | 33 (1.0%) |  |
| Other | 32 (0.4%) | 19 (0.4%) | 13 (0.4%) |  |
| Occupational category |  |  |  | <0.01 |
| Professional-Managers | 2,424 (32.3%) | 1,403 (32.7%) | 1,021 (31.7%) |  |
| Higher-grade white collar workers | 2,011 (26.8%) | 1,199 (28.0%) | 812 (25.2%) |  |
| Lower grade white collar workers | 1,778 (23.7%) | 1,048 (24.5%) | 730 (22.7%) |  |
| Independent workers | 185 (2.5%) | 100 (2.3%) | 85 (2.6%) |  |
| Blue collar workers | 660 (8.8%) | 340 (7.9%) | 320 (10.0%) |  |
| Not concerned | 267 (3.6%) | 120 (2.8%) | 147 (4.6%) |  |
| Other | 176 (2.3%) | 75 (1.8%) | 101 (3.1%) |  |
| Living situation |  |  |  | <0.01 |
| Couple with children (own or partner's) | 3,224 (42.9%) | 1,831 (42.7%) | 1,393 (43.3%) |  |
| Couple without children | 2,019 (26.9%) | 1,211 (28.2%) | 808 (25.1%) |  |
| Single parent with children | 537 (7.2%) | 310 (7.2%) | 227 (7.0%) |  |
| Living alone | 1,204 (16.0%) | 691 (16.1%) | 513 (15.9%) |  |
| Cohabiting with others (family, friends, roommates, etc.) | 410 (5.5%) | 202 (4.7%) | 208 (6.5%) |  |
| Other | 28 (0.4%) | 10 (0.2%) | 18 (0.6%) |  |
| Unknown | 87 (1.2%) | 34 (0.8%) | 53 (1.6%) |  |
| BMI category |  |  |  | 0.90 |
| Underweight | 214 (2.9%) | 128 (3.0%) | 86 (2.7%) |  |
| Normal weight | 4,378 (58.4%) | 2,501 (58.4%) | 1,877 (58.4%) |  |
| Overweight | 2,136 (28.5%) | 1,217 (28.4%) | 919 (28.6%) |  |
| Obese | 773 (10.3%) | 440 (10.3%) | 333 (10.4%) |  |
| Smoking status |  |  |  | <0.01 |
| Current smokers | 1,219 (16.2%) | 646 (15.1%) | 573 (17.8%) |  |
| Former smokers | 2,252 (30.0%) | 1,282 (29.9%) | 970 (30.1%) |  |
| Never smokers | 4,034 (53.7%) | 2,359 (55.0%) | 1,675 (52.0%) |  |
| Unknown | 4 (0.1%) | 2 (0.0%) | 2 (0.1%) |  |
| Alcohol consumption status |  |  |  | 0.12 |
| Never | 796 (10.6%) | 488 (11.4%) | 308 (9.6%) |  |
| Monthly or less | 2,776 (37.0%) | 1,562 (36.4%) | 1,214 (37.7%) |  |
| Weekly | 3,105 (41.4%) | 1,771 (41.3%) | 1,334 (41.4%) |  |
| Daily or more | 823 (11.0%) | 463 (10.8%) | 360 (11.2%) |  |
| Unknown | 9 (0.1%) | 5 (0.1%) | 4 (0.1%) |  |

Variables defined as: sex(male, female, other); age group (18-39, 40-64, 65+ years); education (primary, secondary, tertiary); living situation (living alone, couple without/with children, single parent); nationality (Swiss vs. non-Swiss). Work occupation was defined as (Professional-Managers, Higher-grade white-collar workers, Lower grade white-collar workers, Independent workers, Blue collar workers, Not concerned, Other). Work situation was defined as the current employment status (Salaried, Retired, Freelance/sole trader, Homemaker, Student/training, Unemployed, Long-term disability, Other). Financial situation was defined with a self-assessment: "Average to Poor" when participants self-reported they cannot meet needs or covers basic needs; "Good/Very Good" when participants self-reported they can cover needs with ability to save. Body Mass Index category (World Health Organization classification) was defined as (Underweight, Normal weight, Overweight, Obese). Smoking status was defined as (current smoker, former smokers, never smokers and unknown); alcohol consumption status was defined as (Never, monthly or less, weekly, daily or more).

*^1^* n (%); Mean (SD), n=15 were removed from the analysis because of missing data regarding their alcohol consumption.*^2^* Pearson’s Chi-squared test; Wilcoxon rank sum test; Fisher’s exact test
